# Insects Use Two Distinct Classes of Steps during Unrestrained Locomotion

**DOI:** 10.1371/journal.pone.0085321

**Published:** 2013-12-23

**Authors:** Leslie M. Theunissen, Volker Dürr

**Affiliations:** 1 Department of Biological Cybernetics, Bielefeld University, Bielefeld, Germany; 2 Cognitive Interaction Technology - Center of Excellence, Bielefeld University, Bielefeld, Germany; Imperial College London, United Kingdom

## Abstract

**Background:**

Adaptive, context-dependent control of locomotion requires modulation of centrally generated rhythmic motor patterns through peripheral control loops and postural reflexes. Thus assuming that the modulation of rhythmic motor patterns accounts for much of the behavioural variability observed in legged locomotion, investigating behavioural variability is a key to the understanding of context-dependent control mechanisms in locomotion. To date, the variability of unrestrained locomotion is poorly understood, and virtually nothing is known about the features that characterise the natural statistics of legged locomotion. In this study, we quantify the natural variability of hexapedal walking and climbing in insects, drawing from a database of several thousand steps recorded over two hours of walking time.

**Results:**

We show that the range of step length used by unrestrained climbing stick insects is large, showing that step length can be changed substantially for adaptive locomotion. Step length distributions were always bimodal, irrespective of leg type and walking condition, suggesting the presence of two distinct classes of steps: short and long steps. Probability density of step length was well-described by a gamma distribution for short steps, and a logistic distribution for long steps. Major coefficients of these distributions remained largely unaffected by walking conditions. Short and long steps differed concerning their spatial occurrence on the walking substrate, their timing within the step sequence, and their prevalent swing direction. Finally, ablation of structures that serve to improve foothold increased the ratio of short to long steps, indicating a corrective function of short steps.

**Conclusions:**

Statistical and functional differences suggest that short and long steps are physiologically distinct classes of leg movements that likely reflect distinct control mechanisms at work.

## Introduction

Adaptability of locomotion is a prerequisite for natural autonomous behaviour in an unpredictable environment. In legged locomotion, adaptability is known to involve mechanical and sensory feedback in addition to centrally generated rhythmic motor patterns [1–4]. Although much is known about distinct neural and biomechanical mechanisms underlying adaptive phenomena, it remains largely unknown whether and how these distinct mechanisms affect step-to-step variability under real-world conditions. In order to fill this gap, it will be necessary to understand the natural statistics of unrestrained locomotion for two reasons: First, the natural variability of kinematic and dynamic key parameters will reveal the natural range and frequency of proprioceptive information and, thus, chart the behaviourally relevant sensory input to various sensory-driven mechanisms. Second, the knowledge about how continuous or discontinuous the key parameters of stepping vary during locomotion will shed light on how the underlying mechanisms may be modulated (continuously) or recruited (discontinuously).

With this in mind, the objective of this study is to understand how spatial parameters of unrestrained stepping are affected by spatial irregularities of the substrate. For this, we recorded the whole-body kinematics of unrestrained walking and climbing insects as we systematically varied the height of two stairs on an otherwise flat walkway. More specifically, our goal was to identify invariant features of step length distributions, and to understand how the variable features of these distributions differ between leg types, how they depend on the behavioural context (e.g., the climbing effort), and how they are affected by reduced tarsal grip.

Adaptation of step length may depend on a number of sensory modalities. Humans adjust their leg positions according to visual feedback [5,6] and they adapt to differences in the terrain by adjusting step length, by lifting their legs higher or by changing direction (reviewed in [7]). Additionally, several task-, phase- and context-dependent reflexes are integrated for preserving balance and ensuring stable walking (reviewed in [8]). In cats, the combination of centrally generated rhythms and sensory feedback helps to preserve balance when a hind leg steps into a hole [9,10]. The resultant correction movements depend on prior experience, posture and the speed of locomotion [9] and they are enhanced by supra-spinal pathways [10]. In insects, sensory feedback involves visual [11,12], tactile [13,14] and proprioceptive cues [15,16]. This feedback is thought to act on circuits generating rhythmic motor patterns [4,17]. Despite the wealth of physiological insight into the interplay of (predictive) centrally generated and (reactive) sensory feedback mechanisms in motor control, our view of the behavioural relevance of this interplay still is largely based on the integration of various results obtained on restrained animals, rather than on empirical investigations of unrestrained locomotion.

In this study, we chose the stick insect *Carausius morosus* (de Sinéty, 1901), which is a long-standing model organism for the analysis of multi-legged locomotion [16], to study whole-body coordination during unrestrained walking and climbing. As a starting point, we attended to step length and step direction as key parameters of locomotor adaptability. Stick insects may adapt both of these parameters, e.g. for maintaining spatial coherence of stepping by means of a targeting mechanism [18,19], during turning [20,21] or in response to antennal contact [13]. Furthermore, a number of more or less anecdotal reports on various insect species have described the occurrence of conspicuously short steps. Most of these reports have suggested them to be a result of step length modification in response to a spatial disturbance. For example, stick insects (*Aretaon asperrimus*) execute relatively short steps when climbing across large gaps [22]. An ethogram analysis indicated an increased occurrence of such short steps close to the leading edge of the gap. Similarly, short steps have been described to occur in cockroaches (*Blaberus discoidalis*) that climb high obstacles, where they are used to place their tarsi closer to the obstacle [23]. Also, locusts (*Locusta migratoria*) have been reported to use short steps when climbing across obstacles and overcoming ditches [24]. The latter study coined the term “local searching movements” for repetitive short steps, occurring when walking on rough terrain. Repetitive short steps were also observed in stick insects (*Carausius morosus*), when the tarsi were covered by paint [25]. Given these observations, it was remained to be tested how short steps may contribute to adaptive locomotion and, in particular, whether they are part of a continuum of steps with variable step length or rather represent a distinct class of steps. In the first case, it would be reasonable to assume a continuous mechanism of step length regulation. In the latter, a discontinuous recruitment mechanism of a distinct step class would have to be postulated. Finally, we were curious to find out whether short steps also occurred during unperturbed walking along a flat path. In this case, the underlying mechanism may not be triggered by an external disturbance but may reflect a competitive process among step generating mechanisms.

Our analysis focuses on step parameters rather than gait parameters. This is for four reasons: First, gaits are mainly defined as patterns of temporal coordination across the entire body [26], neglecting spatial coordination. As yet, natural environments are rich in spatial disturbances that are bound to affect spatial step parameters such as step length and step direction. Second, gaits may be subject to continuous fluctuation, leading to a large percentage of episodes with no gait being clearly identifiable. For example, Dürr demonstrated highly variable gaits in stick insects, even for very similar walked paths [27]. Similarly, Grabowska et al. recorded “undefined” gaits in 36 to 66% of episodes of walking in stick insects, depending on substrate slope [28]. As a consequence, quantifying subtle changes in gait is an unsolved problem. Third, legs differ functionally [29], even in animals with morphologically similar leg pairs such as stick insects. Therefore, the proprioceptive feedback will differ among legs participating in the same gait. Based on these considerations, it appears easier to identify indicators of distinct sensory-motor control mechanisms in a single leg than across an entire body. Finally, in insect locomotion, a single leg may be “taken out” of the stepping pattern by placing it on a platform while the other legs are engaged in ongoing locomotion [30]. This and other findings indicate that the insect walking system consists of a set of coupled single-leg controllers rather than one central six-leg controller (for a de-centralised model of adaptive walking see [31,32]). In this view, a gait arises through coupling of adjacent single-leg controllers and adaptations in gait are caused by adaptations of at least one single-leg controller.

Here, we used a motion capture system to record unrestrained locomotion of intact animals and animals with manipulated substrate engagement. Step parameter distributions were drawn from a recording period of 2 hours including several thousand steps. We find that the natural step length distribution is always bimodal, and that the two modes have distinct functional and statistical properties. Furthermore, we confirm the significance of foothold through experimental manipulation, and conclude that stick insects take two distinct classes of steps during locomotion: long steps and short steps. We argue that these step classes indicate distinct underlying control mechanisms for propulsion and correction of inappropriate foothold, respectively.

## Materials and Methods

### Behavioural experiments

For the experiments, 23 adult female stick insects of the species *Carausius morosus* (de Sinéty, 1901) were used [33]. Animals were bred in a laboratory culture at Bielefeld University. For the duration of the experiments, they were kept at a minimum temperature of 21°C.

**Figure 1:**
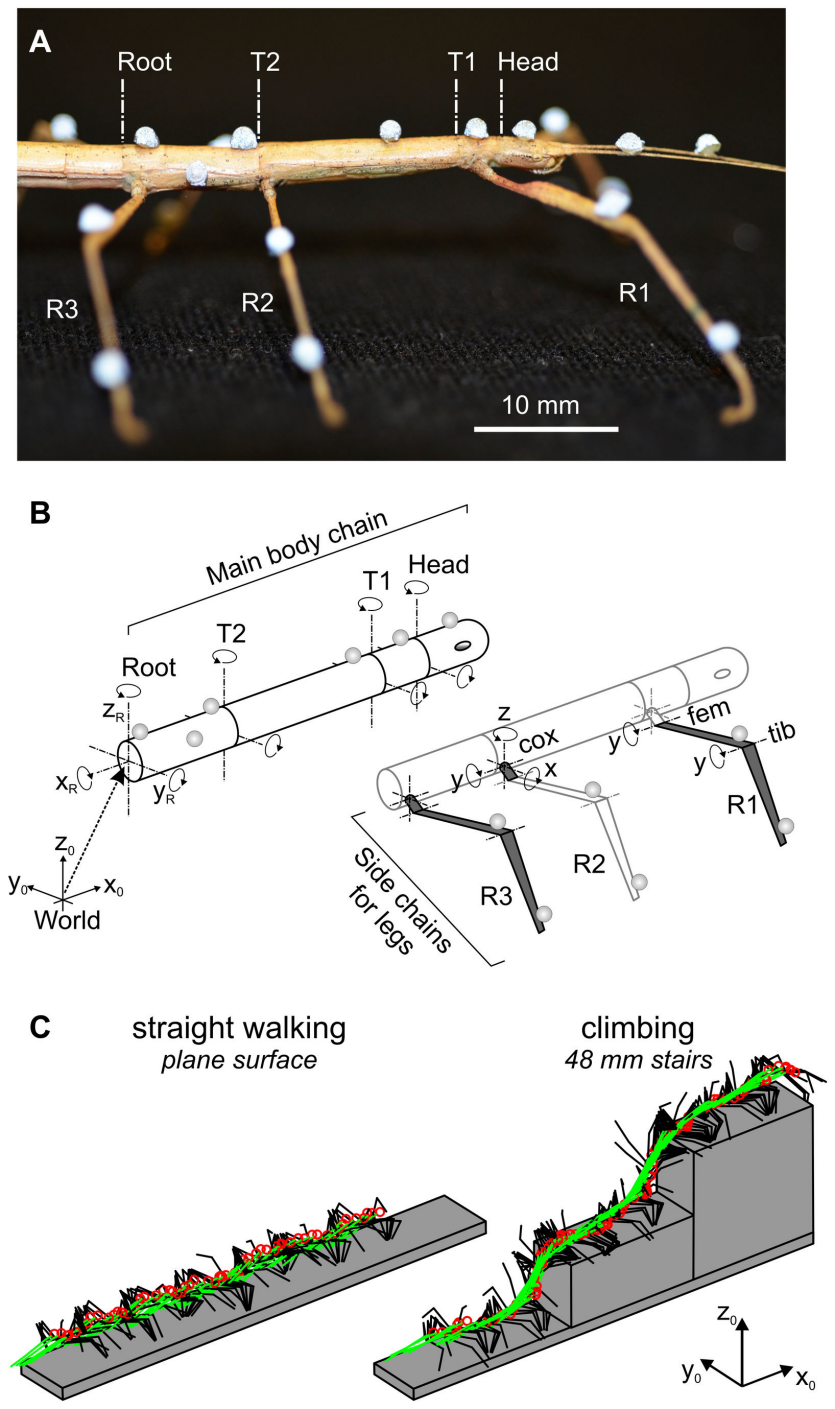
A marker-based motion capture technique was used. A: Insects were labelled with reflective markers. B: For kinematic analysis, the body was modelled as a branched kinematic chain. The main body chain (left) consisted of the three thorax segments (Root, T2, T1) and the head. Six side chains (right) modelled the legs, with the segments coxa, femur and tibia (cox, fem, tib; only right legs are shown, labelled R1 to R3). All rotation axes (DoF) are indicated (3 for the root segment, 2 for thorax/head segments, and 5 per leg). DoF are denoted according to the subsequent segment and the axis of the local coordinate system around which the rotation was executed. Leg DoF were: cox.x, cox.y, cox.z (labelled for R2), fem.y and tib.y (labelled for R1). C: Two of the four conditions, with stick figures drawn every 100 ms, showing the body axis (green *lines*), the head (red *circle*s) and both front legs (black lines). *Left*: Flat walking condition without stairs. *Right*: High climbing condition with 48 mm stairs.

In each experimental trial, an animal was placed on a horizontal walkway (40 x 490 mm; polyvinyl chloride), along which it walked freely (Figure 1C). Four walking/climbing conditions were presented in a randomised sequence of at least 40 trials: in the *flat* (walking) condition (Figure 1C, left panel), the walkway was used without stairs; in the climbing conditions *low*, *middle* and *high* (Figure 1C, right panel), a staircase with two stairs of step height, h, was placed at the end of the walkway (40 x 200 mm; *low*: h = 8 mm, *middle*: h = 24 mm, *high*: h = 48 mm). The *flat* walking condition served as the reference condition. The *low* stairs were at the normal body height above ground during horizontal walking; *middle* stairs were in reach of one high swing movement; *high* stairs required foot placement on the vertical wall. The whole setup was painted in opaque black and was surrounded by black drapery in order to minimise visual contrast. The room was darkened and illuminated only by red light LEDs of the Vicon cameras (see below) and indirect light emanating from a TFT computer monitor. 

A marker-based motion capture technique was applied, for which each animal was labelled by 17 or 18 retro-reflective markers with a diameter of 1.5 mm (Figure 1A). Markers were glued to the cuticle by use of transparent nail polish. Two markers were attached to each leg, one to the distal femur and one to the distal tibia (Figure 1B, right panel). Additionally, five markers were attached to thorax and head, with three markers defining the body-fixed coordinate system of the metathorax and one additional marker on the prothorax and head (Figure 1B, left panel). In most animals, a further marker was placed on the rostral mesothorax. Care was taken that neither the nail polish nor the markers constrained the movement of any joint. Finally, a body model was established for each animal, consisting of a branched kinematic chain with a four-segmented body axis and six three-segmented limbs. For this, segment dimensions and the positions of all markers on their respective body segment were measured from high-resolution photographs (0.02 mm per pixel) taken under a stereo lens (Olympus SZ61T, equipped with a Pixelink camera PL-B681CU, controlled by µScope software).

A Vicon MX10 motion capture system with eight T10 cameras (Vicon, Oxford, UK) was used for data acquisition of marker positions. Temporal resolution of the motion capture system was 200 Hz; spatial resolution was approximately 0.1 mm. The time of entry of the animal into the capture volume was used as starting frame of the recording. The recording was stopped when the animal reached the far end of the setup. Trials were discarded if the animal climbed the side walls of the setup instead of the stairs, or stopped walking before the first stair. In this case, the same trial condition was repeated. One animal always executed double steps with its right hind leg, where each step was followed by a brief and short swing movement. This animal was excluded from the analysis, because its behaviour was clearly different from that of the other nine intact animals. An additional digital video camera (Basler A602fc, Ahrensburg, Germany) equipped with a near range zoom lens (Edmund Optics, Barrington, NJ, USA) was used to record a complementary image sequence for visual inspection, e.g., for validation of the kinematic analysis. The video showed a side view of the climbing sequence of the first stair, with a temporal resolution of 50 Hz (synchronized with the Vicon system) and a spatial resolution of approximately 0.14 mm per pixel. The software Nexus 1.4.1 (Vicon, Oxford, UK) was used for controlling the motion capture process and for subsequent offline analysis. Each of the markers was identified and labelled once by hand. Markers were then tracked automatically, provided that each marker was recorded by at least two cameras. The resulting trajectories of spatial coordinates of all markers were inspected for filling of small trajectory gaps. Generally, marker detection was very robust. On average, less than 5 gaps per 60 s occurred in single marker trajectories, with mean trial durations of 11.29 ± 4.8 s (equivalent to 2258 ± 964 frames; mean ± s.d.). Gaps shorter than 200 ms (40 frames) were filled by use of an interpolation algorithm of the software Nexus.

### Whole-body kinematics

Calculation of the whole-body kinematics from recorded marker trajectories was necessary for two reasons: (I) for determining time and location of touch-down and lift-off events for each foot, and (II) for calculation of step length within a body-centred coordinate system (CS, see below). Whole-body kinematics yielded the joint angle time courses associated with 42 degrees of freedom of motion (DoF) of the body model. With regard to the body-centred CS, all joint positions were expressed in right-handed Cartesian coordinates, with the x-axis pointing rostrad within the sagittal plane, i.e., from the origin towards the head, the horizontal y-axis pointing towards the left within the horizontal body plane, and the z-axis pointing dorsad within the sagittal plane. All calculations were done in Matlab (The MathWorks, Natick/MA, USA), using the toolbox *c3dserver* (Motion Analysis Laboratory, Erie, PA, USA) for importing C3D data from Vicon Nexus.

#### Scaling and filtering

Joint angles were calculated by use of two data sets coming from (I) the segment lengths and marker positions on the animal, as calibrated under the stereo lens, and (II) the marker trajectories, as obtained from motion-capturing. Since the body model measurements were more precise than the Vicon calibration, the marker trajectories were scaled by the factor l_BM_/l_MC_, where l_BM_ is the distance of two markers in the body model with fixed distance (e.g., two markers on the metathorax), and l_MC_ is the corresponding mean distance of the same markers in the motion capture data. l_BM_/l_MC_ ranged from 0.94 to 1.00, mainly depending on the calibration quality of the Vicon system. After scaling of marker trajectories, the time courses of all marker coordinates were low-pass filtered in Matlab, using a 4^th^ order Butterworth filter with a cut-off frequency of 20 Hz.

#### Calculating the main body chain

The main kinematic chain included the three thorax segments and the head (Figure 1, left panel). The root segment (metathorax, including the fused 1^st^ abdominal segment) had six DoF: three translational DoF indicating the position of the body in the external coordinate frame [x_0_, y_0_, z_0_] and three rotational DoF indicating roll, pitch and yaw rotation around the x_0-_, y_0-_ and z_0_-axis, respectively. The other three segment joints of the main body chain had two rotational DoF each: pitch and yaw rotation around the segments y- and z-axes, respectively. This resulted in twelve DoF for the main chain. In four animals with 17 markers (without second mesothorax marker), the metathorax-mesothorax joint was assumed to be immobile.

The rotation of the root segment with respect to the world coordinate system was determined from the axis orientations of a body-fixed root coordinate system ([x_R_, y_R_, z_R_] in Figure 1B). The latter was defined by the three markers on the root segment, such that x_R_ pointed in the direction of the main chain and z_R_ was orthogonal to the plane defined by the three markers. The calibration images of the side marker on the root segment yielded a bias rotation angle. Back-rotating the marker-fixed root coordinate system by this angle aligned [x_R_, y_R_, z_R_] with the sagittal, horizontal and frontal body planes. Measures taken from calibration images were then used to determine the origins of all connecting segments. In case of the root segment, these were the mesothorax (T2) and the hind leg coxae. Next, the vector connecting the root-T2 joint with the marker on T2 was calculated. After back-rotating this vector by its bias rotation with respect to [x_R_, y_R_, z_R_], as determined from calibration images, its polar coordinate angles yielded the joint rotation angles around the axes T2.z and T2.y. The resulting T2-fixed coordinate system was used to calculate the origins of the prothorax (T1) and of the middle leg coxae. The rotation angles of the T2-T1 joint and T1-head joint, along with the remaining segment origins of the main body chain were calculated in analogy to the calculation steps taken for T2.

#### Calculating the six side chains

Each thorax segment connected to two kinematic side chains, modelling the left and right legs (see Figure 1C, right panel, where R1 to R3 label the right front to hind legs). The side chains consisted of a coxa with three rotational DoF in the thorax-coxa joint (ThC-joint, [protraction/retraction, levation/depression, supination/pronation]), the trochantero-femur (subsequently called femur) with one DoF in the coxa-trochanter joint (CTr-joint, [levation/depression]), and the tibia with one DoF in the femur-tibia joint (FTi-joint, [extension/flexion]). For calculation of the leg joint angles, the first step was to determine the “leg plane” spanned by the two leg markers and the origin of the corresponding side chain. If the normal vector of this plane was expressed within the coordinate system of its connecting thorax segment, its polar coordinate angles gave the protraction/rectraction and supination/pronation of the ThC-joint, along with the rotated z- and x-axes defining the leg plane. The sum of levation/depression in the ThC- and CTr-joints was then calculated by expressing the vector connecting the ThC-joint to the femur marker within the xz-coordinate system of the leg plane. From the known segment lengths of coxa and femur, along with the exact marker position on the femur, the relative contribution of the ThC- and CTr-joint to femoral levation could be determined by triangulation. Finally, the known femur length was used to determine the location of the FTi-joint, and the vector connecting the latter to the tibia marker was used to calculate the extension/flexion of the FTi-joint (with consideration of the bias rotation caused by the misalignment of the tibial marker and the tibial axis). 

### Foot contacts and step length

Step length was defined as the Euclidean distance covered by the tibia-tarsus joint, i.e., the proximal end of the foot, during a swing movement. According to this definition, a step started at the point of lift-off and ended at the point of touch-down. We calculated the step length in body-fixed coordinates, accounting for differences of body orientation during walking and climbing. To differentiate between stance and swing phases, the time courses of all foot (tibia-tarsus joint) positions were smoothed a second time, using a first-order low-pass filter with a cut-off frequency of 66.6 Hz. Next, foot velocity [mm/s] was calculated as the distance covered between two frames. The following criteria were used to find foot contact positions. If the velocity was below 25 mm/s for at least eight consecutive frames (40 ms; equivalent to an average distance of less than 1 mm), the foot was considered as standing still. These phases were only interpreted as stance phases, if the distance between mean foot position and substrate was less than 5 mm. This plausibility constraint excluded very slow leg movements without substrate contact. To transform the foot contact positions into body-fixed coordinates, we used the thorax-fixed root CS at the instant when the leg touched down or lifted off, respectively. For analysis of step parameter distributions of each leg type (i.e., front, middle and hind legs), steps of left and right legs were pooled together, after inversion of the left leg’s y-coordinate. Statistical analyzes were calculated with the Matlab statistics toolbox. For the circular analysis, the “Toolbox for circular statistics” was used [34]. In the experiments with intact animals, the number of animals was always nine.

### Ablation experiments

For testing a hypothesis about the role of foothold and/or grip, we manipulated the grip of the right middle leg tarsus by ablating only the claw and the arolium (Figure S1A; N = 8 animals) or the entire distal tarsal segment (Figure S1B; N = 5). Ablations were done manually with a razor blade under a stereo lens and documented by photographs. Wounds were sealed with wax. The experimental procedure was essentially the same as for the intact animals, except that only the *flat* and the *high* condition were used (see Figure 1C). For the analysis, we used the intact left middle leg as a within-subject comparison for the effect of ablation. 

## Results

Stick insects readily walked along the walkway and generally showed no difficulty in climbing the stairs in the three climbing conditions (Figure 1C). At first sight, climbing of low stairs looked like walking in the *flat* condition, with no apparent change in walking speed, body posture, or leg movement. With increasing height of the stairs forward velocity was reduced, body posture was inclined, and increasingly more steps were placed on the vertical walls or on the edges of the stairs. We analysed 323 trials from nine intact animals (33 to 40 trials per animal), including a total of 17307 steps. On average, this amounted to 10.3 steps per front leg, 9.5 steps per middle leg and 8.9 steps per hind leg per trial. Compared to flat walking trials, the step number per trial increased with the height of the condition by 15 to 19% on low stairs, by 37 to 43% on middle stairs, and by 39 to 44% on high stairs. This increase cannot be explained by the increase in walking distance alone, which increased only by approximately 5% on low, 16% on middle and 32% on high stairs. Rather, animals tended to take more and shorter steps during climbing than during walking on plane surface. To investigate this in more detail, we compared the relative frequency distributions of step length between conditions.

### Stick insects take two classes of steps

**Figure 2:**
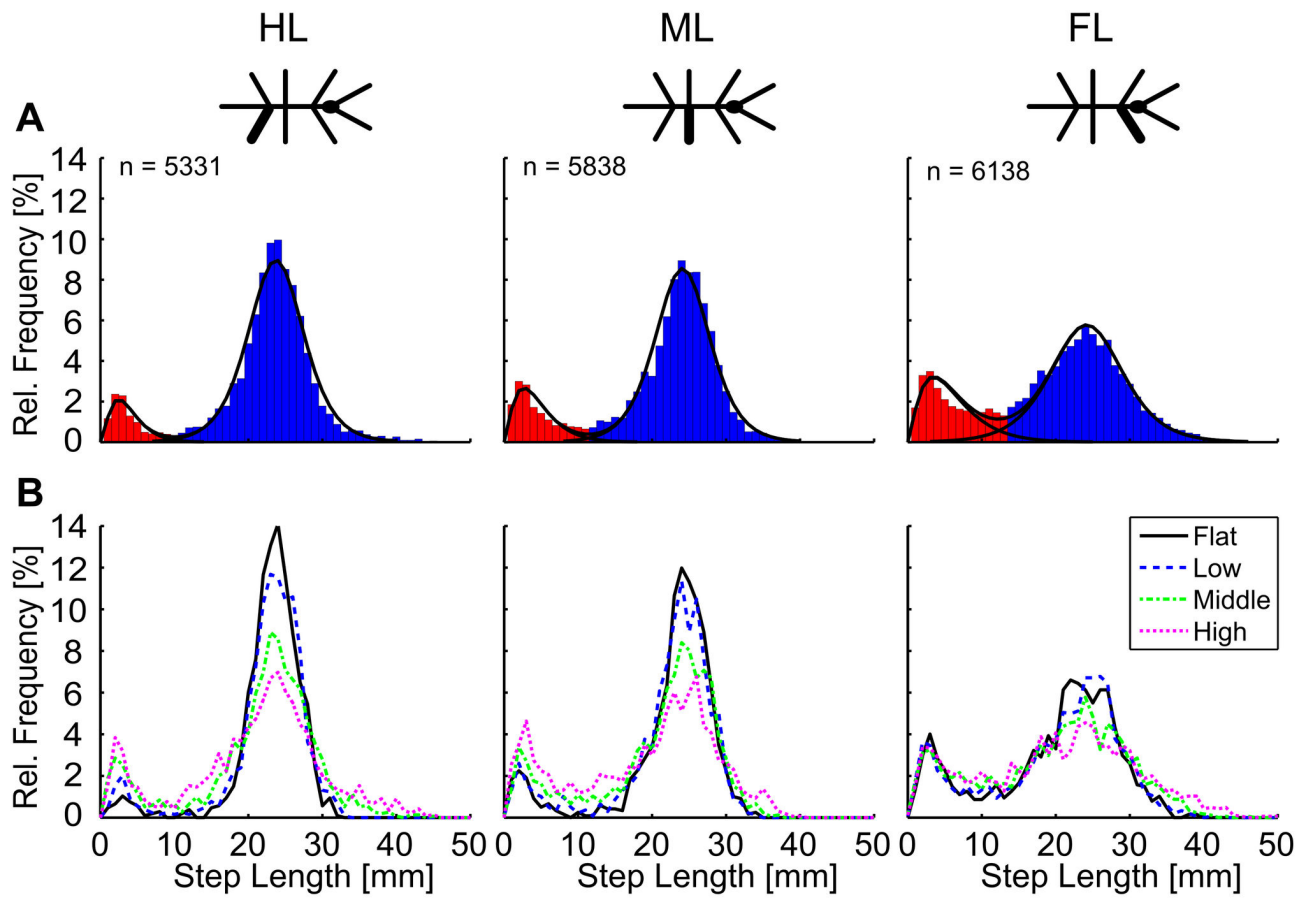
Step length distributions were always bimodal. Relative frequency of step lengths for hind legs (HL), middle legs (ML), and front legs (FL) always had two distinct local maxima. Steps of right and left legs were pooled for legs of the same segment. A: Data pooled from four walking/climbing conditions. Red and blue bars indicate short and long step classes, respectively, assigned according to their location relative to the local minima of the distributions (Local minima: FL 13 mm; ML 11 mm; HL 10 mm). Two curve fits and their sum are superimposed: a gamma distribution function and a logistic distribution function were fitted to the short and long steps, respectively. B: Distributions of the same data as above, but separated for the four walking/climbing conditions. Note how the local maxima remain at the same location, irrespective of walking condition. Sample numbers for the four conditions (flat, low, middle, and high) were: n_FL_ = (1312, 1547, 1839, 1821); n_ML_ = (1215, 1406, 1679, 1716); n_HL_ = (1146, 1365, 1625, 1579).

Distributions of the step length were always bimodal, i.e., showing two distinct peaks, independent of leg type and trial condition (Figure 2A). For pooled samples, the first peak appeared at a step length of 2 mm in middle and hind legs (ML, HL), and 3 mm in front legs (FL), whereas the second peak appeared at 23 mm in HL and 24 mm in FL and ML. Based on this observation, the distributions were subdivided into *short steps* and *long steps* (red and blue areas in Figure 2A), using the local minimum as the border between the two classes of steps. This resulted in different relative frequencies of short steps in the three leg types (FL: 25%, ML: 16%, HL: 10%). When comparing the properties of the two modes with those of a normal distribution, it became evident that the first mode was much more asymmetrical, and the second mode was considerably more narrow than a normal distribution. To account for these differences, we found that the probability density functions of a Gamma and a Logistic distribution provided the best fit to the empirical distributions. A gamma distribution with *f*(*x*|*a,b*) = 1/(*b* ^*a*^Γ*(a*))·*x* ^*a*-1^ *e* ^-x/b^ was used for the short steps, where *Γ*(*a*) is the gamma function and *a*, *b* are parameters setting the rate of increase and decay. A logistic distribution with *f*(*x*) = *e* ^(x-µ^)^/σ^/(σ(1+*e* ^(x-µ)/σ^)^2^) was used for the long steps, where µ is a parameter of central tendency and σ > 0 is a parameter of dispersion. The parameters of the best curve fits are given in Table 1, separated by trial condition. The results show that the peak location of the step length probability distributions were affected only little by trial condition (µ ranged within only 1.2, 0.8 and 0.2 mm, *a* ranged within 0.3, 0.3, and 0.8 in FL, ML and HL, respectively). Furthermore, there was a monotonous tendency of the long steps to become more variable in length with increasing height of the stairs (see parameter σ in Table 1; distributions differ in spread in Figure 2B). The main effect of trial condition on step length distribution was a change in proportion of short steps over long steps, which increased with increasing height of the stairs (Figure 2B). This change in proportion was clearest in ML and HL distributions, which were similar and which showed a lower variability than FL distributions. The fact that the two classes of any step length distribution were well-described by distinct probability distributions, the central tendency (i.e., peak location) of which remained largely unaffected by trial condition, supports the hypothesis that stick insects take two distinct classes of steps: short and long steps.

**Table 1:** Parameters of probability distributions fitted to data in Figure 2.

	FL				ML				HL			
	a	b	µ (+)	σ (+)	a (+)	b	µ	σ (+)	a	b	µ	σ (+)
Pooled	2.3	2.6	24.2	3.2	2.4	1.9	24.1	2.5	2.8	1.4	23.7	2.5
Flat	2.4	2.4	23.7	2.7	2.5	1.3	24.4	1.9	2.9	1.4	23.7	1.7
Low	2.2	2.6	24.0	2.8	2.5	1.6	24.1	2.1	3.2	1.3	23.8	1.8
Middle	2.3	2.8	24.3	3.4	2.7	1.7	24.0	2.6	2.3	1.7	23.9	2.7
High	2.5	2.5	24.9	4.0	2.8	1.6	23.6	3.3	2.4	1.8	23.7	3.5

The gamma distribution function that was fitted to the short steps is characterized by *a* and *b*, and the logistic distribution function that was fitted to the long steps is characterized by µ and σ. All parameters are given for front (FL), middle (ML) and hind legs (HL) with respect to pooled data (Figure 2A), and to fits to the different conditions (*flat*, *low*, *middle* and *high*). Parameters exhibiting a small monotonic increase with the height of the stairs are labeled with (+).

**Figure 3:**
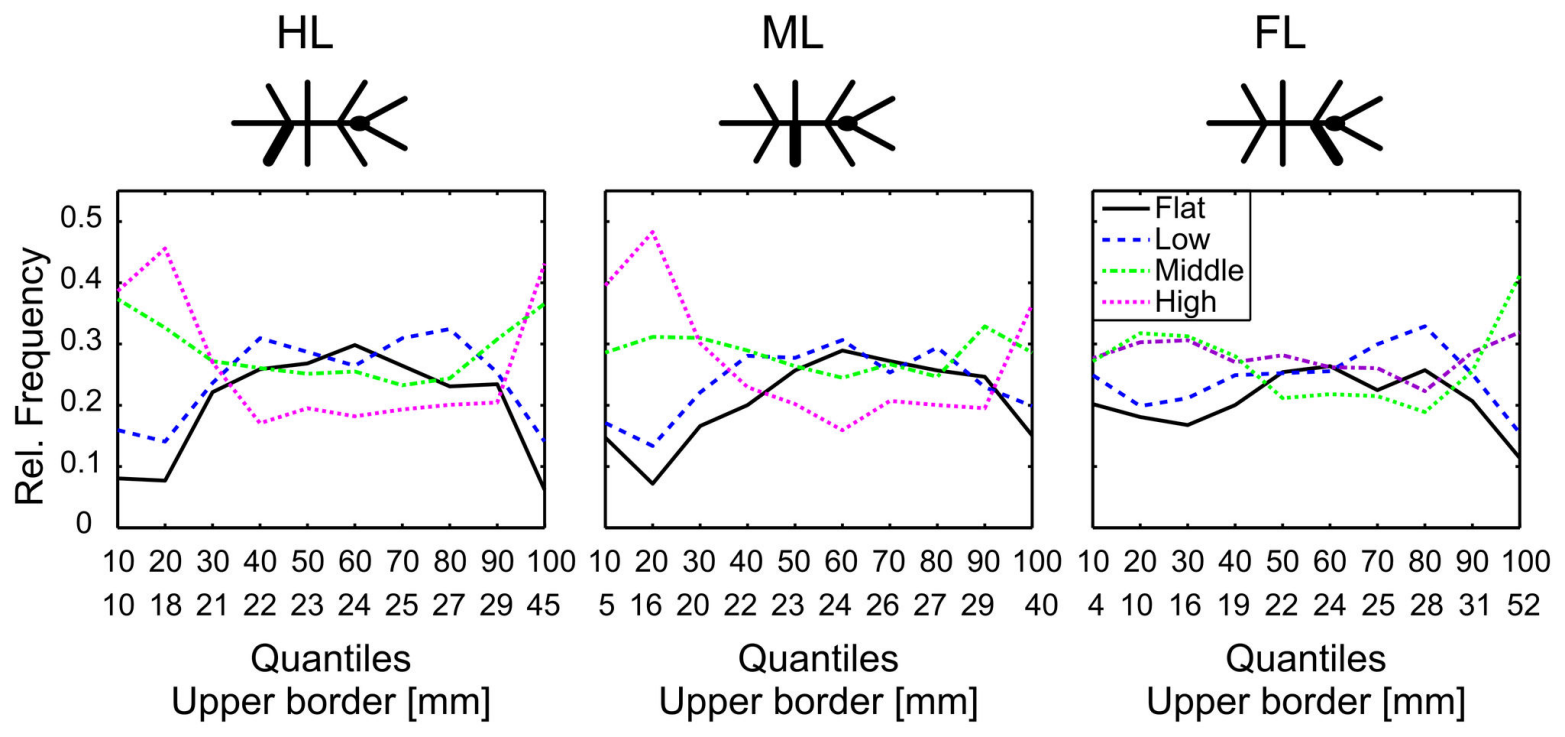
Proportion of steps of given length differs with walking condition. Proportions are given for 10% quantiles of the distribution of all steps (see Figure 2A). Numbers below abscissa indicate quantiles (e.g. 10: 1 to 10%; 20: 11 to 20%). Numbers below quantiles indicate the upper borders of step length comprised by the corresponding quantile. The figure shows that the proportion of short and very long steps increases with the height of the stairs. In contrast, steps of intermediate length show no systematic difference. Number of steps in each quantile: n_FL_ = 663/664; n_ML_ = 613/614; n_HL_ = 576/577.

In relation to their absolute numbers, we wanted to understand the proportions of short and long steps in the different climbing conditions. Therefore, we first subdivided the step length into 10% quantiles and then divided the steps according to the four conditions (*flat*, *low*, *middle* and *high*). The differentiation showed two local maxima of the *high* condition (Figure 3), i.e. the numbers of short steps (quantiles < 20%) and of very long steps (quantile 100%) were highest for the *high* condition and decreased with the decreasing height of the stairs. During walking on a plane (Figure 3, *flat*), stick insects most often took long steps. Steps of intermediate length were similarly distributed across the different conditions and showed no systematic difference, whereas short and very long steps appeared more frequently during climbing conditions. This result matched the different heights of the stairs, where each condition included parts of horizontal walking, and the animals had to take the largest steps to reach the edge of the high stairs. However, the stairs did not explain steps of about 2 mm length. As stick insects took more short steps in climbing trials, we wanted to know where and when these steps occurred. Specifically, we wanted to distinguish among the following three different hypotheses: (I) Short steps are used before climbing, for getting closer to the vertical wall; (II) they are used during climbing, for finding appropriate foothold (i.e., substrate engagement); or (III) they are used on top of the stairs, for maintaining stability and for correction of an inconvenient leg position. 

### Short and long steps are not evenly distributed

**Figure 4:**
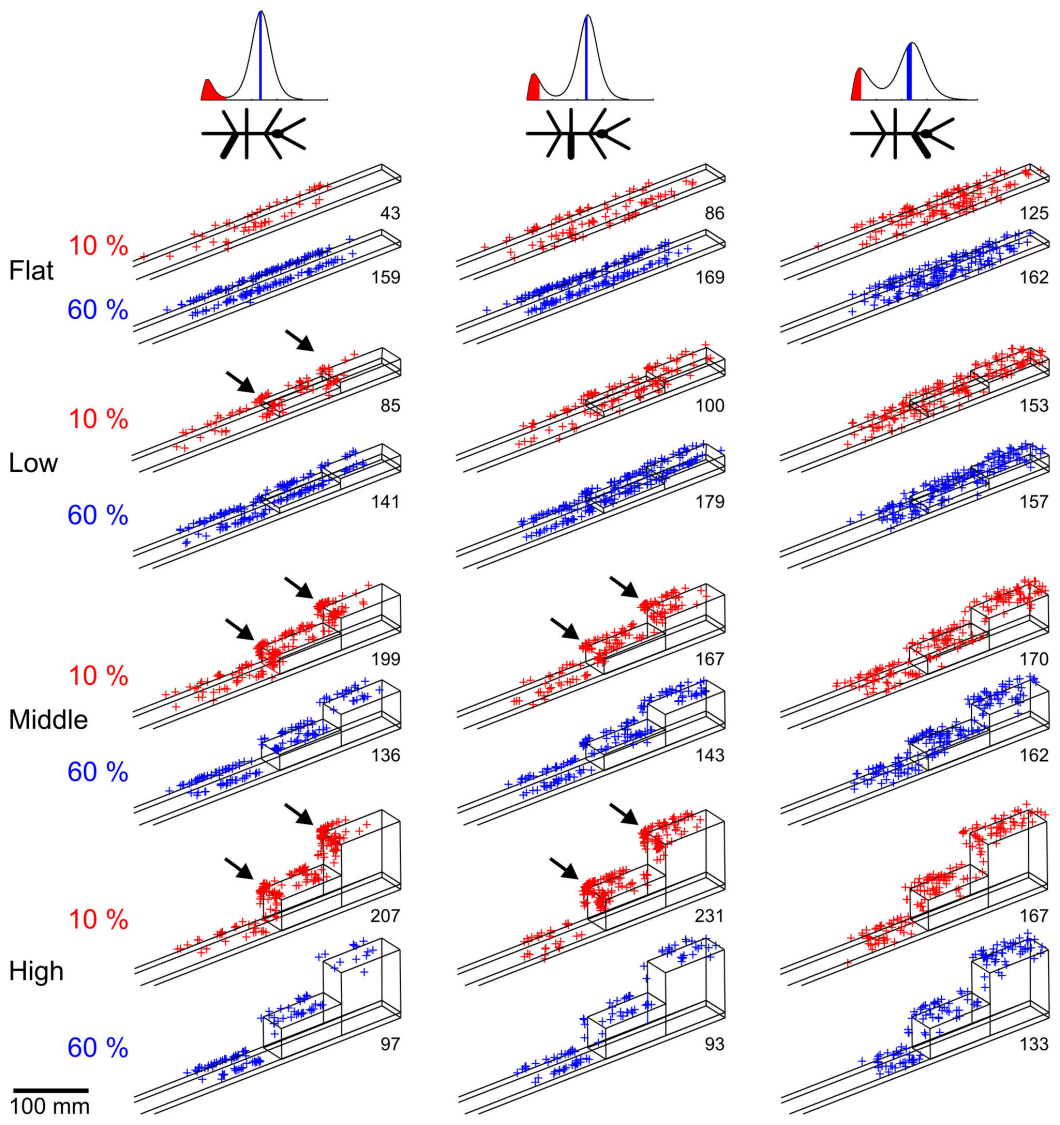
Short steps cluster around edges of the setup. Touch-down positions on the setup for hind, middle and front legs. The positions (crosses) are shown for different conditions for short and long steps. Short steps (red) are represented by the shortest 10% of the step length distribution. Long steps (blue) are represented by the median range (50 - 60%). The lines show the setup in each condition. In case of the climbing conditions (middle and high), short steps cluster at the edges of the setup (arrows). The numbers of steps (n) are given below each plot.

To analyze the touch-down locations, we chose two sub-samples of the entire distribution, each one comprising 10% of the total number of steps per condition. Short steps were represented by the 0% to 10% percentiles of the distribution and long steps were represented by the mean length (50-60% percentiles of the distribution). Figure 4 shows the touch-down positions on the setup of these two samples. In the *flat* condition, no clustering was observed and all step types were evenly distributed across the whole setup. In the two climbing conditions (*middle* and *high*), the ML and HL touch-down positions of short steps clustered around the edges of the staircases (Figure 4, arrows). Some short steps occurred in front or on top of the vertical walls, but most of them were located on the vertical walls. The high density of touch-down positions of short steps during climbing mirrors the increasingly larger proportion of short steps with increasing height of the stairs (Figure 2B). This effect was more pronounced in ML and HL than in FL. In contrast to the touch-down locations of short steps, the touch-downs of long steps (Figure 4, blue crosses) were distributed equally across horizontal surfaces in all trial conditions, but less on the vertical walls. This may indicate their use in propulsion on horizontal surfaces. The distributions of touch-down positions of short steps show that they were predominantly executed during climbing. Furthermore, stick insects placed their legs most often close to the lateral side of the setup (Figure 4). This is reasonable, because the width of the walkway (40 mm) corresponded to the distance of stick insect’s left and right feet when walking on a plane. Nevertheless, short steps could be used to place the legs in the middle of the walkway. To test this hypothesis, we compared the lateral distribution of touch-downs of short and long steps and could not find any significant difference (Kolmogorov-Smirnov test, N = 9, FL/ML/HL: p = 0.80/0.91/0.54, n_long_ = 4585/4915/4788, n_short_ = 1560/923/543, k = 0.09/0.09/0.12). Therefore, we can exclude that short steps were used to adjust the medio-lateral location of foot contact with the walkway.

### Short and long steps differ with respect to timing

**Figure 5:**
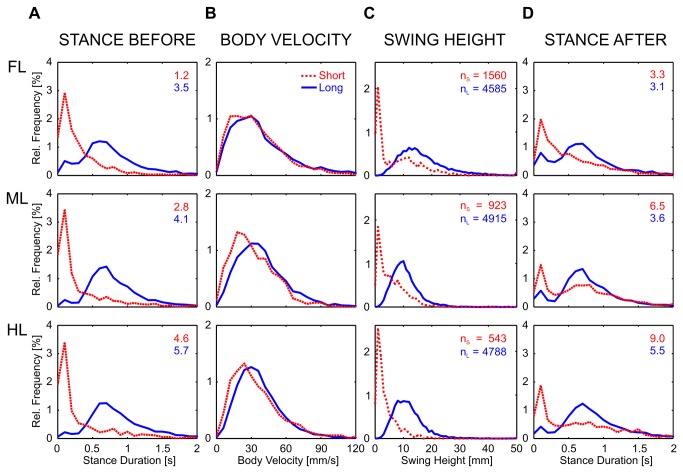
Body velocity, swing height and stance duration before and after the swing phase differ between short and long steps. The relative frequencies are shown for long (blue) and short (red) steps for each leg type (rows). A: The stance duration before swing is the stance phase immediately preceding the swing movement of the reference step. Stance durations were binned to 0.1 s and cut off at 2 s, with the percentage of longer stance phases given by the number to the upper right. B: Body velocity is the movement velocity of the body origin when a leg lifted off the ground. C: Swing height represents the height range of the tarsus during swing phase, binned to 1 mm bins. The total numbers of steps is given in C (n). D: Stance duration after swing is the stance phase immediately following touch-down.

Knowing the particular spatial distribution of short steps, we wondered whether their temporal properties, particularly their timing within the step sequence, differed from long steps as well. Furthermore, we compared swing height and body velocity at the time of lift-off between the two classes of steps. Observations of single trials showed that double-stepping was frequent during climbing. To understand whether or not short steps were used as the second step of such double-stepping, we analyzed stance duration immediately before and after swing phases of short and long steps (Figure 5A,D). Stance duration was defined as the duration from touch-down to lift-off. Figure 5A shows that the stance duration preceding a long step was significantly longer than that preceding a short step (Kolmogorov-Smirnov test, FL/ML/HL: p < 0.001/0.001/0.001, n_long_ = 4585/4915/4788, n_short_ = 1560/923/543, k = 0.56/0.65/0.64). The latter showed a peak at 0.1 s indicating that most short steps occurred shortly after the preceding step. For the stance phases following the reference step (Figure 5D), this peak remained at 0.1 s, but it was smaller, and stance phases following short steps were significantly longer than those preceding short steps (Kolmogorov-Smirnov test, FL/ML/HL: p < 0.001/0.001/0.001, n_short_ = 1560/923/543, k = 0.23/0.41/0.31). For example, there was an increase in the proportion of stance durations longer than 2 s in HL, from 4.6 to 9.0%, whereas the proportion of such long stance durations slightly decreased for long steps (Figure 5A,D).

In stick insects, stance duration varies with walking velocity [29,35]. Therefore, we were interested at which velocities the different step classes occurred predominantly. Figure 5B shows that the body velocity was reduced when the leg lifted off for a short step (Kolmogorov-Smirnov test, FL/ML/HL: p < 0.005/0.001/0.001, n_long_ = 4585/4915/4788, n_short_ = 1560/923/543, k = 0.05/0.16/0.11). Nevertheless, short steps could occur at all walking velocities, excluding the possibility that they were a consequence of slow walking. The proportion of long steps at very low velocities was small, which indicates that long steps drive propulsion. 

Short and long steps differed in their spatial occurrence, their timing and their association with high/low walking velocities. To understand their functional properties, we next analyzed the swing movements in more detail. First, we looked at the swing height, which was defined as the dorsal-ventral range of the tibia-tarsus joint during swing phase in body-fixed coordinates. In level walking, this is equivalent to the maximum tarsus height above ground. The relative frequencies of swing height of the two classes of steps are shown in Figure 5C. During the swing phases of long steps, the tarsus covered an average height range of 14.8 mm, 9.8 mm and 10.8 mm for FL, ML and HL, respectively (quartiles: 25% = 10.7/7.3/7.9 mm, 75% = 20.3/12.7/14.1 mm). Whereas the average height range of short steps was only 4.7 mm, 4.2 mm and 2.6 mm, respectively (quartiles: 25% = 1.1/1.6/1.2 mm, 75% = 12.5/8.1/5.8 mm). The distributions of swing height of short steps showed a peak at 1 mm for all leg types (Figure 5C) and differed significantly from the distributions of long steps (Kolmogorov-Smirnov test, FL/ML/HL: p < 0.001/0.001/0.001, n_long_ = 4585/4915/4788, n_short_ = 1560/923/543, k = 0.47/0.48/0.70). As for the step length, FL showed a wider distribution in swing height than ML and HL. Not surprisingly, swing duration was also shorter in short steps than in long steps (data not shown). In summary, during a short step stick insects lifted their legs neither as high nor as long as during a long step.

### What is the function of short steps?

**Figure 6:**
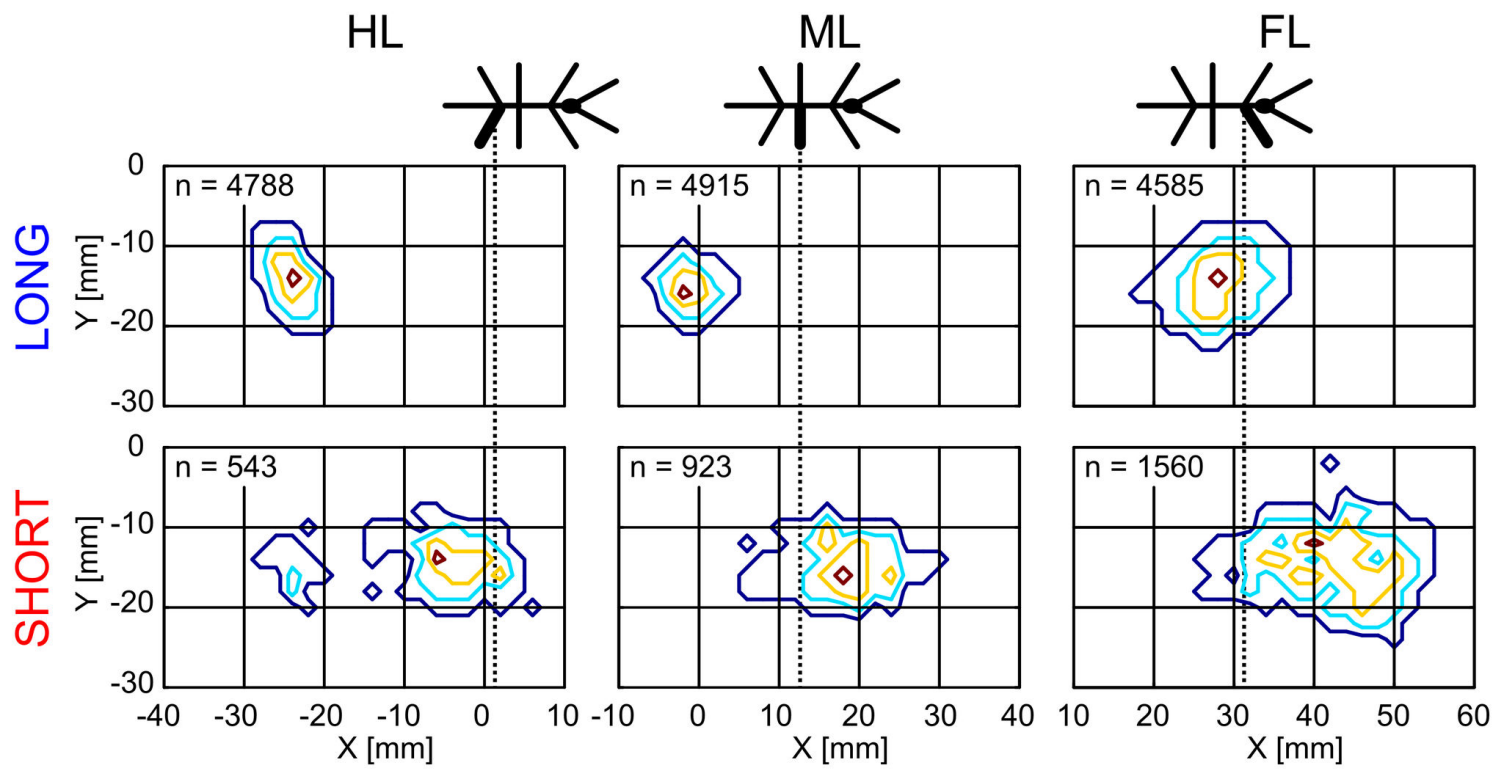
Short steps lift off more anteriorly than long steps. Top view of the 2D-probability distributions of lift-off positions in body-fixed coordinates. The lines indicate percentile ranges (red: max, yellow: 25%, turquoise: 50% and blue: 75%). The origin (0/0) was set to the metathorax-abdomen border. The dashed lines indicate the mean positions of the corresponding thorax-coxa joints.

During rhythmic stepping, swing movements have the function to return the foot to the position, where the propulsive stance phase (power stroke) begins. In case of interspersed short steps, the function of a swing movement appeared to be different. The hypothesis that short steps were predominantly used for getting closer to the vertical wall could be rejected because most touch-down locations were near the edges of the stairs (Figure 4). The two alternative hypotheses that short steps serve to correct for either inappropriate foothold, i.e., substrate engagement, or inconvenient leg position remain to be solved. If correcting for inappropriate foothold, we expected that lift-off positions were close to the preceding touch-down position, mainly because substrate engagement is achieved at the beginning of stance [36]. As a result, we expected that short steps would lift off more anteriorly than “regular” long steps. Indeed this is what we found. For both classes, lift-off positions of all leg types were widely spread (Figure 6). Lift-off positions of short steps were located more anterior than those of long steps (Mann-Whitney U test, FL/ML/HL: p < 0.001/0.001/0.001, n_long_ = 4585/4915/4788, n_short_ = 1560/923/543, z = 45.29/45.25/31.66). Most of the short steps of FL and ML lifted off even more anteriorly than the ThC-joint of the corresponding leg, indicating a protracted ThC-joint angle. According to the reasoning above, the anterior lift-off positions suggested that short steps corrected for an inappropriate foothold, following a short period of ground contact. We assumed that a correction for inappropriate foothold could be undirected, meaning without having a preferred swing direction. In contrast, we reasoned that an inconvenient leg posture may result from imprecise long steps and would likely be corrected by swinging into a position-dependent direction. For example, if the leg touched down too laterally, the correction should go medially. In comparison, swing movements of long steps should be directed rostrally, at least if they were to be followed by a propulsive stance phase.

**Figure 7:**
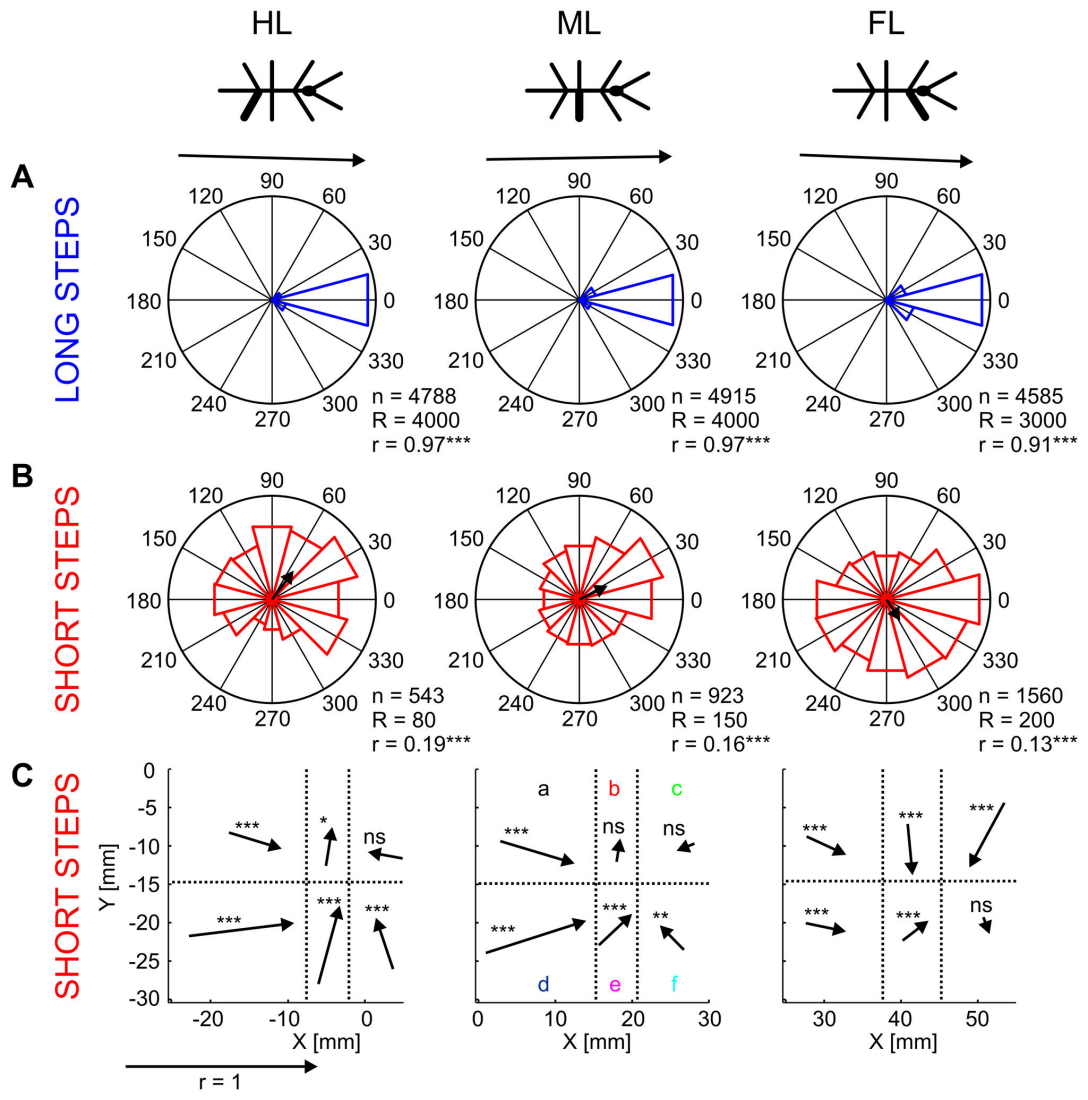
Step direction differs between short and long steps. A, B: Circular histograms for long and short steps. Step direction was calculated from lift-off to touch-down in the horizontal plane of body-fixed coordinates. The directions match the orientation of the stick figure on top. The arrows on top (long steps) and in the middle of the histograms (short steps) show the mean direction vectors. The number of steps (n), the radius (R) of the circle and the length of the mean direction vector (r) are given to the lower right of each plot. C: Mean direction vectors of short steps separated according to lift-off positions. The lift-off positions were subdivided into 6 fields (a to *f*) according to quantiles (33.3% and 66.7% for x, and 50% for y; dotted lines). For each of the fields, the mean direction vector was calculated. Note, that the lengths of the arrows do not correspond to the mean step lengths. Instead, all arrows in A, B and C are scaled relative to the arrow shown below the graph, which has the length of r = 1. The asterisks indicate the level of significance of Rayleigh-tests (ns: not significant, * p < 0.05, ** p < 0.01, *** p < 0.001 for more details see Table S1).

Based on these considerations, we analyzed swing direction in short and long steps. For this, we calculated the angle between lift-off and touch-down as projected onto the subjective horizontal plane in body-fixed coordinates (XY-plane). Figure 7 shows circular distributions of step directions for long (Figure 7A) and short steps (Figure 7B) of each pair of legs. Apart from few exceptions, long steps were exclusively directed rostrally. Accordingly, mean directions of long steps were highly significant (Rayleigh test, FL/ML/HL: p < 0.001/0.001/0.001, n_long_ = 4585/4915/4788, z = 3756/4606/4544). The corresponding mean direction vectors reached 91 to 97% of resultant length, indicating nearly perfect alignment with the body axis. In contrast, swing directions of short steps were more widely distributed, and mean direction vectors differed between leg type, being directed rostro-laterally, almost rostrally, and rostro-medially in FL, ML, and HL respectively (mean directions FL: 299.0; ML: 24.4; HL: 55.2; Rayleigh test, FL/ML/HL: p < 0.001/0.001/0.001, n_short_ = 1560/923/543, z = 26.61/24.91/19.07). Note that, despite statistically significant mean directions, short steps could be directed into any direction, including backwards (reflected by very short resultant vectors in Figure 7B and by the low z-values). The latter never occurred in long steps. Our analysis of the directionality was unaffected by the accuracy of the motion capture system. This was shown in control experiments, where we added a constant error value to the lateral deviation and calculated the swing direction of short and long steps (Figure S2). The result shows that a systematic error could not account for the omnidirectional distribution of short step direction. 

Next, we analysed whether the broad distribution of step direction in short steps was related to potential proprioceptive cues, such as joint angles. As joint angles at lift-off depend on foot position, we investigated the mean direction vectors for six sub-samples of short steps, grouped according to their lift-off position (Figure 7C). Lift-off positions were separated at the median in medio-lateral direction (50%) and at the 33.3% and 66.7% quantiles in rostro-caudal direction. Except for the rostro-medial sample in HL, the central-medial and the rostro-medial samples in ML and the rostro-lateral sample in FL, all mean direction vectors were statistically significant (Rayleigh test: p < 0.05, for details see Table S1), indicating a preferred swing direction. Note that the length of the arrows in Figure 7C does not correspond to step length, but to the consistency of the direction vector (i.e., the shorter the vector, the larger the dispersion). In all leg types, mean direction of short steps depended on lift-off position, always exhibiting a strong rostrad component in caudal samples, a strong mediad component in central samples of HL and ML steps, and a strong laterad component in medial and rostral samples of FL steps. Interestingly, the overall pattern exhibited by the six direction vectors was very similar among leg types, except for the central-medial sample of FL where the mean direction was laterad, while that of the ML and HL was mediad. In the swing movements of ML short steps, radial vector components, i.e., those pointing towards or away from the leg’s ThC-joint, must have been driven by the CTr- and FTi-joint, whereas tangential components must have been driven mainly by the ThC-joint [37]. 

**Figure 8:**
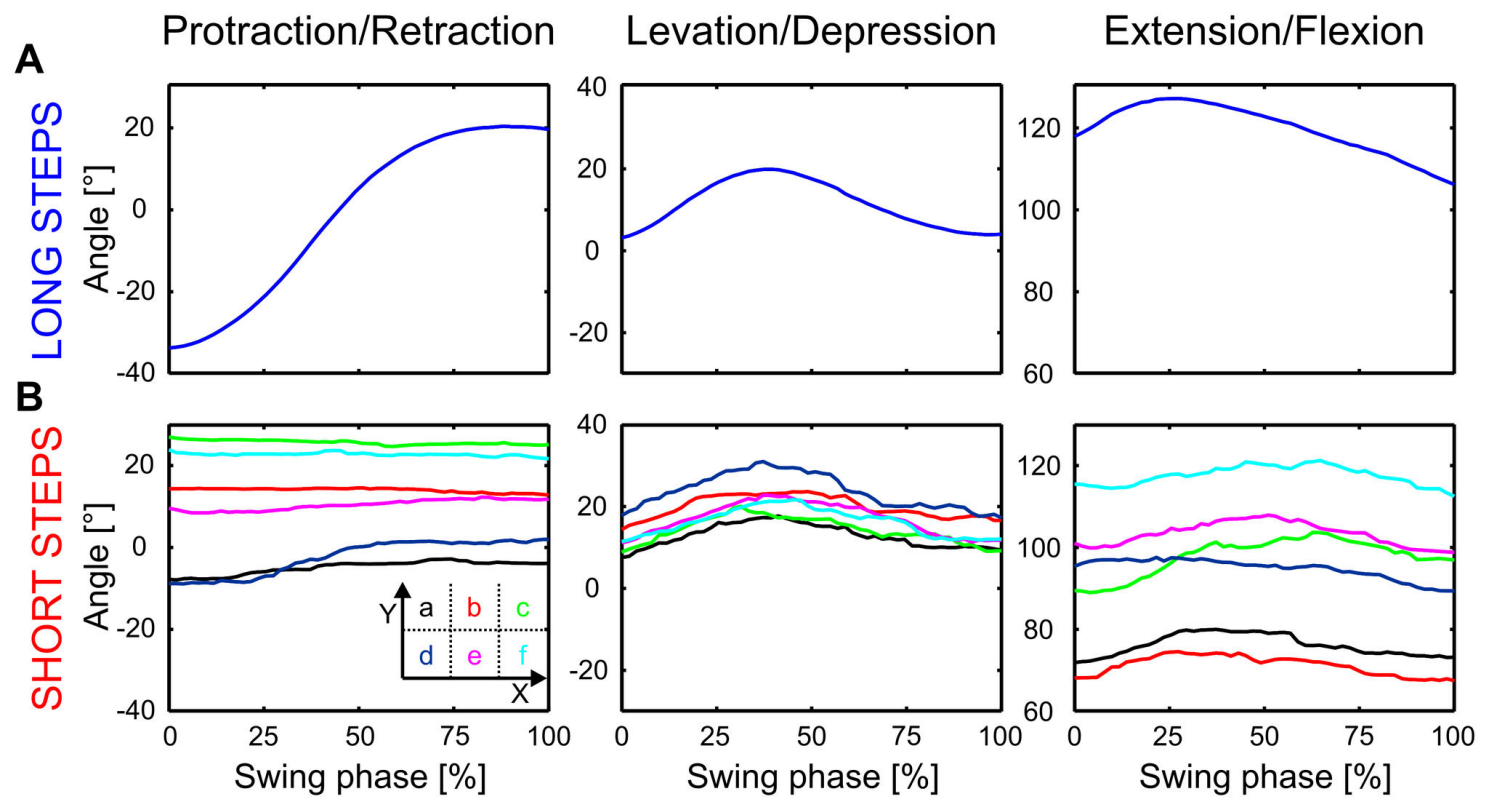
Joint angle time courses of short steps depend on lift-off position. A: Long steps: Median joint angle time courses during the swing movement of the ML, standardized to the mean duration of all swing movements (swing phase in %). Panels from left to right show the protraction/retraction angle of the thorax-coxa joint, the summed levation/depression angles of the thorax-coxa and coxa-trochanter joint, and the extension/flexion angle of the femur-tibia joint. B: Short steps: same plotting details, except that each line corresponds to a different lift-off position (see Figure 7C). Note that, owing to standardization to the mean swing duration, time courses of short steps are stretched and those of long steps are compressed. The number of long steps was 4915. The numbers of short steps of the sub-samples are given in Table S1.

These assumptions were confirmed by the median joint angle time courses of long and short steps of the ML (Figure 8). The rostrad directed long steps always had a strong tangential component and were dominated by a protraction of the ThC-joint, while the leg was elevated and extended in the first half of the swing movement and depressed and flexed in the second half (Figure 8A). The angular ranges of short steps were, as a consequence of short step length and variable swing direction, much smaller than those of long steps. Nevertheless, a clear position dependency was indicated by the different joint angle time courses. Notable protraction was only found in caudal short steps (groups a and d in Figure 8B), and in lateral steps the leg was more extended than in medial steps (a, b, and c, compared to d, e, and f in Figure 8B). Finally, all samples were elevated and extended during the first half of swing and depressed and flexed during the second half. This movement could be used to find appropriate foothold with the tarsus. The position dependency of short steps could be also observed in the joint angle time courses of FL and HL (Figure S3). We conclude that short steps differ functionally from long steps in that their direction is position-dependent with little drive to the ThC-joint unless the leg lifts off from caudal positions.

**Figure 9:**
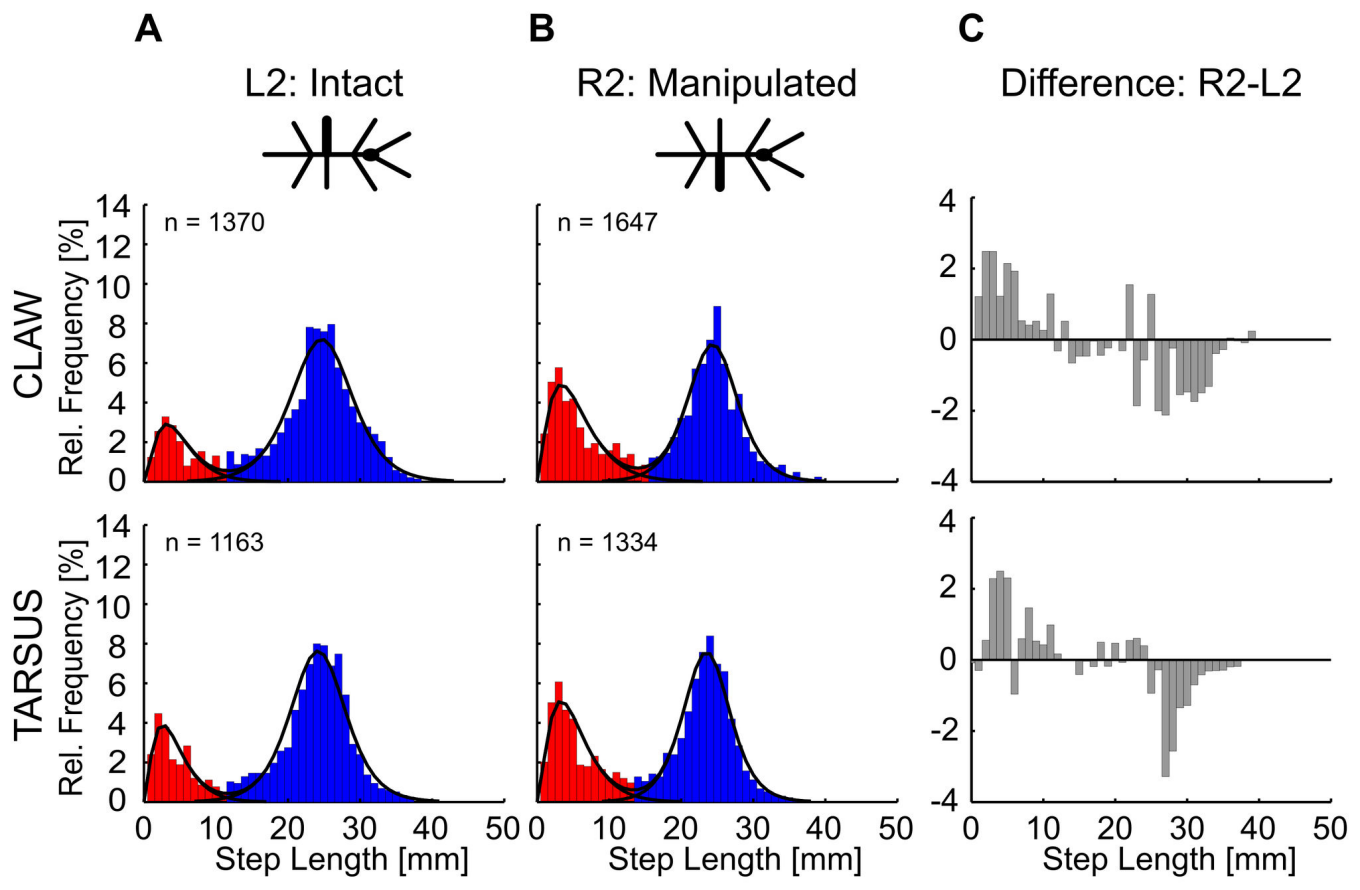
Manipulation of tarsal grip affects the likelihood of short step occurrence. Relative frequencies of step length for intact (L2) and manipulated legs (R2) always had two distinct local maxima, independent of whether the claw (top row) or the fifth tarsal segment (bottom row) was ablated. Red and blue bars indicate short and long step classes, respectively, assigned according to their location relative to the local minima of the distributions. Superimposed curve fits as in Figure 2A. The histograms on the right show the difference of the relative frequency of step lengths between the manipulated and the intact leg, with positive values indicating higher relative frequencies in the manipulated leg.

Whereas the omnidirectionality of short step swing directions was in line with our assumption in case of correction for inappropriate foothold, the slight position-dependency could have resulted from a correction for inconvenient leg position. To provide more evidence for one of these two interpretations, we manipulated the foothold experimentally in two ways: In a first group of animals, we ablated the claw and the arolium of the right middle leg tarsus, leaving all tarsal segments intact, including their adhesive pads (euplantulae) and the apodeme of the retractor unguis muscle (Figure S1). The latter made sure that the tarsus still actively engaged with the substrate. In a second group of animals, we ablated the entire distal tarsomere (5^th^ tarsal segment) and, therefore, cut the apodeme of the retractor unguis muscle. This prevented active contraction of the tarsal segments. In both groups, we compared the step length distribution from the manipulated (right) side with the corresponding distribution from the intact (left) side (Figure 9). We reasoned that, if the frequency of short steps depended on substrate engagement, both manipulations should have led to a left-right asymmetry. In contrast, if the frequency of short steps depended on foot position or other kinematic parameters, no such left-right asymmetry would be expected. After both types of ablation, the operated middle leg executed significantly more short steps compared to its intact neighbour (χ^2^-tests for contingency of treatment and step class; claw: d.f. = 1, p < 0.001, χ = 80.9; tarsus: d.f. = 1, p < 0.001, χ = 33.0). After claw ablation, animals executed 24.6% more short steps than expected in case of stochastic independence of treatment and step class. After ablation of the distal tarsal segment, this difference amounted to 16.7%. Since both types of ablation must be expected to have decreased the grip force of the tarsus, the increase in short step occurrence suggests a potential function of short steps as correction for inappropriate foothold.

## Discussion

We investigated the variability of step length and step direction in unrestrained climbing insects. Step length distributions always showed two distinct peaks (Figure 2), revealing two classes of steps in stick insect locomotion: short and long steps. The distributions were well-described by the sum of a gamma and a logistic distribution, of which the major parameters varied little or not at all with context (Table 1). Context, however, did affect the proportion of the two classes of steps, indicating their different function. Short steps clustered at the edges of the stairs (Figure 4), suggesting a function in the control of balance or foothold during climbing. The anterior lift-off positions (Figure 6) and the short preceding stance phases (Figure 5) indicated a corrective function soon after touch-down, as would be expected in case of correction for inappropriate foothold. Moreover, the omnidirectionality of short steps (Figure 7) supports the view that they do not contribute to propulsion. To gain further support for the “correction step hypothesis”, we experimentally impaired substrate engagement of one middle leg tarsus, using two types of ablation. Both types of ablation led to a significant increase in likelihood of short steps in the operated leg, compared to the intact contralateral middle leg (Figure 9), showing that reduced grip and/or substrate adhesion leads to more short steps. The two classes of steps could be controlled by two different mechanisms of step generation, which are context-dependent and influenced by sensory feedback. 

### Spatial coordination and the regulation of step length

Short steps were mentioned in previous studies. For example, *Aretaon asperrimus* takes short steps when approaching large gaps [38]. Similarly, cockroaches use short steps immediately before climbing, to get close to an obstacle [23]. In our study, *C. morosus* adjusted its body orientation when approaching stairs. Therefore, one possibility was that short steps were a means of preparing for climbing, in which case they should have occurred mainly in front of the stairs. As yet, Figure 4 shows that short steps most often touched down on the vertical wall and near the top edge of the stairs. Together with the increasing proportion of short steps with increasing height of the stairs (Figures 2 and 3), this suggests that the function of short steps was particularly relevant during climbing. 

The change in proportion was stronger in HL and ML than in FL. This may be explained by the different function of the legs described by Cruse [29]: When stick insects walk on a horizontal plane, FL often appear to serve as feelers, while ML only support the body weight and HL support body weight and provide thrust for propulsion [29]. When walking on a vertical path, all legs contribute to the support of body weight and to propulsion [29]. In our experiments, the legs probably took on a mix of these functions, as the animals approached the stairs on the plane and then climbed across. The apparent feeler function in the FL is in accordance with the high variability in FL step length and the relatively high proportion of short steps on the plane, compared to the ML and the HL. The finding that short steps were increasingly frequent with increasing time spent in climbing suggests that their recruitment reflects the demand for postural control or acquisition of appropriate foothold. This is because the animals have to sustain their own body weight with their tarsi during climbing.

Step length is known to depend on proprioceptive sensory feedback from coxal hair fields [35]. Ablation of the coxal hair fields leads to longer steps, whereas ablation of trochanteral hair fields, which are thought to be involved in the control of swing height, has no effect on step length [35]. Thus, the information supplied by coxal hair fields should allow the animals to regulate step length for as long as the ThC-joint is involved in the execution of the step. Since short steps often have very little or no mean protraction component (Figure 8B), step length of short steps is unlikely to be regulated by use of coxal hair fields. 

A further mechanism affecting step length in ML and HL is the “targeting mechanism” proposed by Cruse [18] to explain spatial correlation of touch-down positions between ipsilateral leg pairs. Again, proprioceptive hair fields appear to be involved in this spatial coordination of foot placement in stick insects [39,40]. 

### The correction step hypothesis

A strong indicator for the corrective function of short steps is the more anterior lift-off position of short steps compared to that of long steps (Figure 6). Anterior lift-off could be elicited by a reflex that causes the leg to lift off for a short time: the treading-on-tarsus (TOT) reflex [41]. When the front leg tarsus is touched, the TOT reflex causes a short lift-off movement in the ipsilateral middle leg. Efficacy of this TOT reflex depends on the phase of the middle leg’s step cycle and is more likely to be caused when the middle leg is close to its anterior extreme position [41]. This is why we checked for the distance between ipsilateral leg pairs and found that the ML lifted off anterior to the tarsus position of the adjacent front leg in 1.0% of all steps and 7% of short steps. The corresponding criterion for a hind leg was met in 1.7% of all steps and 16.2% of short steps. We concluded that the TOT could explain only a small fraction of short steps. Indeed, single trial inspection of the digital video episodes showed that the TOT reflex was not the prevalent cause for eliciting short steps. In FL, the TOT reflex could not have induced short steps at all. 

Stick insects, as other insect species, carry adhesive pads (euplantulae) on the tarsal segments and claws at the pretarsus (stick insects: [42]; cockroaches: [43,44]). For these structures to function properly, the tarsus needs to adequately engage with the substrate. Therefore, one reason for stick insects taking short steps more often during climbing than during planar walking could be an inappropriate foothold resulting in the lack of substrate engagement. When the animals have to lift their bodies over a stair, as in climbing, each leg can only contribute to the support of the body weight if its tarsus can achieve sufficient grip. In case of lacking grip, i.e., lacking substrate engagement, short steps could serve a corrective function by testing a different touch-down location. This “correction step hypothesis” is supported by the finding that stick insects take a series of short steps with their FL, when the tarsi are covered by paint [25]. Furthermore, Pearson and Franklin [24] qualitatively described “local searching” movements in their experiments on locusts walking on rough terrain. “Local searching” was defined as follows: “Once the leg had located a surface that might be a suitable support, either directly at the end of a normal swing phase or following searching movements and/or an elevator reflex, the tarsus was often moved quickly from point to point on the surface” [24]. They proposed that “local searching” occurs whenever the load on the leg does not quickly increase after the tarsus has touched a surface. This would lead to a fast replacement of the leg to another point. Our findings support this idea, where the leg is rapidly lifted after surface contact, to perform a short step (Figure 5A) and where the joint angle time courses of short steps indicate only little protraction in the ThC-joint, but always a flexion in the FTi-joint to the end of swing (Figure 8B). Several sensor types at the insect tarsus are known that could measure this missing increase in load [45,46]. One such group of sensors are campaniform sensilla. Indeed, cockroaches use tarsal campaniform sensilla to detect substrate engagement [36]. Tarsal campaniform sensilla can be found in *C. morosus*, too (Schmitz, unpublished observation). Additionally, tibial campaniform sensilla encode for forces and could be used to detect ground contact or slipping [47]. Such load signals seem to be weighed against other signals from the legs [17] and could initiate short corrective steps. Finally, we showed that ablation of the tarsal claws or ablation of the entire distal tarsomere increased the proportion of short steps in the manipulated leg (Figure 9). Both structures aid substrate engagement [36,48], adding further support to the “correction step hypothesis”.

### Temporal coordination and the role of stability

Appropriate temporal coordination is important for maintaining balance. This is even more relevant during climbing, when each leg contributes to the support of the body weight. As short steps become more frequent in climbing episodes, an alternative to the “correction step hypothesis” could be that short steps serve to stabilize the body posture, i.e., for maintaining balance by adjusting the leg position. If this was the case, short steps should have occurred more frequently in situations with few feet having ground contact. However, at the time of lift-off of a short step, four or five legs had ground contact in most cases (FL: 76%; ML: 79%; HL: 70%). Since balance should be stable with four or more legs on the ground, lack of balance cannot explain the occurrence of short steps. Related to this issue, it has been argued that after ablation of an entire middle leg – a situation much less stable than in intact walking - leg coordination is not adapted because of reduced stability but because of missing sensory information from the ablated leg [35].

Short steps often followed a stance period of 0.1 s duration (Figure 5A). Therefore, the “decision” whether the substrate contact is an appropriate foothold must be very fast. In cockroaches, the elasticity of the tarsus is used for fast engagement and disengagement from substrates [49]. The muscular arrangement of the claws in stick insects is similar and they are capable of fast tarsus movement, too [48]. This is necessary to perform short steps, where the leg has very short ground contact and rapidly is lifted again (Figure 5A). The particularly short stance period and the small step length favour interspersing of short steps into a nearly regular temporal coordination of stepping. During flat walking, the six legs are well coordinated and, for example, the touch-down of a hind leg favours the initiation of a swing movement in the ipsilateral middle leg [50]. But the information transfer from one leg to the next takes time. Approximately 0.3 s after the touch-down of a hind leg, the ipsilateral middle leg makes a swing movement with a likelihood of 60% [27]. This period is long enough to perform a short step in between, without affecting the stepping pattern during flat walking. This agrees with findings in cockroaches, where the centrally generated rhythmic motor activity in adjacent legs seems to be affected less by single, irregularly timed movements, which occur near the beginning or the end of a burst cycle [51] – reminiscent of short steps.

The temporal coordination of the six legs is linked to the control of walking velocity, such that a tetrapod coordination is more likely during slow walking, whereas a tripod coordination is more likely in faster walking [1,35,52]. Stick insects increase walking velocity by reducing the duration of the stance phases, while keeping swing durations constant [35]. For that reason, short stance phases indicate increased walking velocity. Our results show that in case of short steps the opposite was the case: the velocity of the body was usually slower at lift-off of a short step than it was at lift-off of a long step (Figure 5B). The distribution of stance durations following short steps was wider than preceding short steps. Nevertheless, short steps were often followed by short stance durations, indicating that they could occur consecutively (Figure 5D), as observed by Rosano and Webb [25]. One reason for the lower velocity during short steps could be that most of them were taken during climbing, when the velocity was reduced. 

## Conclusions

Our results show that stick insects take two distinct classes of steps during locomotion. These two classes of steps, separated by the step length, differ in their spatial occurrence, their timing and their functional properties. Whereas long steps are equally placed across the whole walkway, short steps cluster at the edges of the stairs. Short steps most often occur after very short, uncompleted, stance phases and, therefore, they may be interspersed within the normal temporal coordination of the six legs. We propose that missing or insufficient increment in the load signals from the leg may be the cause for executing a short step. Long steps clearly contribute to propulsion. In contrast, we propose that short steps are correction steps that occur in instants of insufficient or inappropriate substrate engagement of a foot. 

## Supporting Information

Figure S1 **Tarsus manipulation experiments.** A tarsus consists of five tarsomeres (numbered from proximal to distal) and the claws and the arolium. We either cut off the claw and the arolium (red line in A) or the entire fifth tarsomere (red line in B). Dots indicate unsclerosed membranes.
(TIF)

Figure S2 **Limited position accuracy had no influence on the directional distribution of long and short steps.** Same graphic details as in Figure 7A,B. To calculate the control of the direction angles, the x-value in body-fixed coordinates is kept and the y-value is replaced by random values of the range of the maximal jitter between the two thorax-fixed markers of all trials (= 0.6544). The number of steps (n) and the radius (R) of the outer circle are given on the lower right of each plot.
(TIF)

Figure S3 **Joint angle time courses differ between FL (top) and HL (bottom), and between short steps (second and fourth row from top) and long steps (first and third row from top).** Same plot details as in Figure 8, but for different leg types. The number of long steps was 4585 for FL and 4788 for HL. The numbers of short steps of the sub-samples are given in Table S1.
(TIF)

Table S1 **Statistically relevant parameters of the mean swing directions of short steps, grouped according to their lift-off positions.** Short steps were separated as described in Figure 7C into six sub-samples (*a* to *f*). The table shows the number of steps (n) of each sub-sample, which was used to calculate the mean swing direction (Figure 7C) and the median joint angle time course in Figure 8B (ML) and in Figure S2B,D (FL, HL). The level of significance (p) and the z-value correspond to the Rayleigh test used to calculate the mean swing directions.
(DOCX)
