# Supplementary figures and images for: Insects Use Two Distinct Classes of Steps during Unrestrained Locomotion
Source: PLoS One. 2013 Dec 23;8(12):e85321. doi: 10.1371/journal.pone.0085321 (PMC3871641; doi:10.1371/journal.pone.0085321)

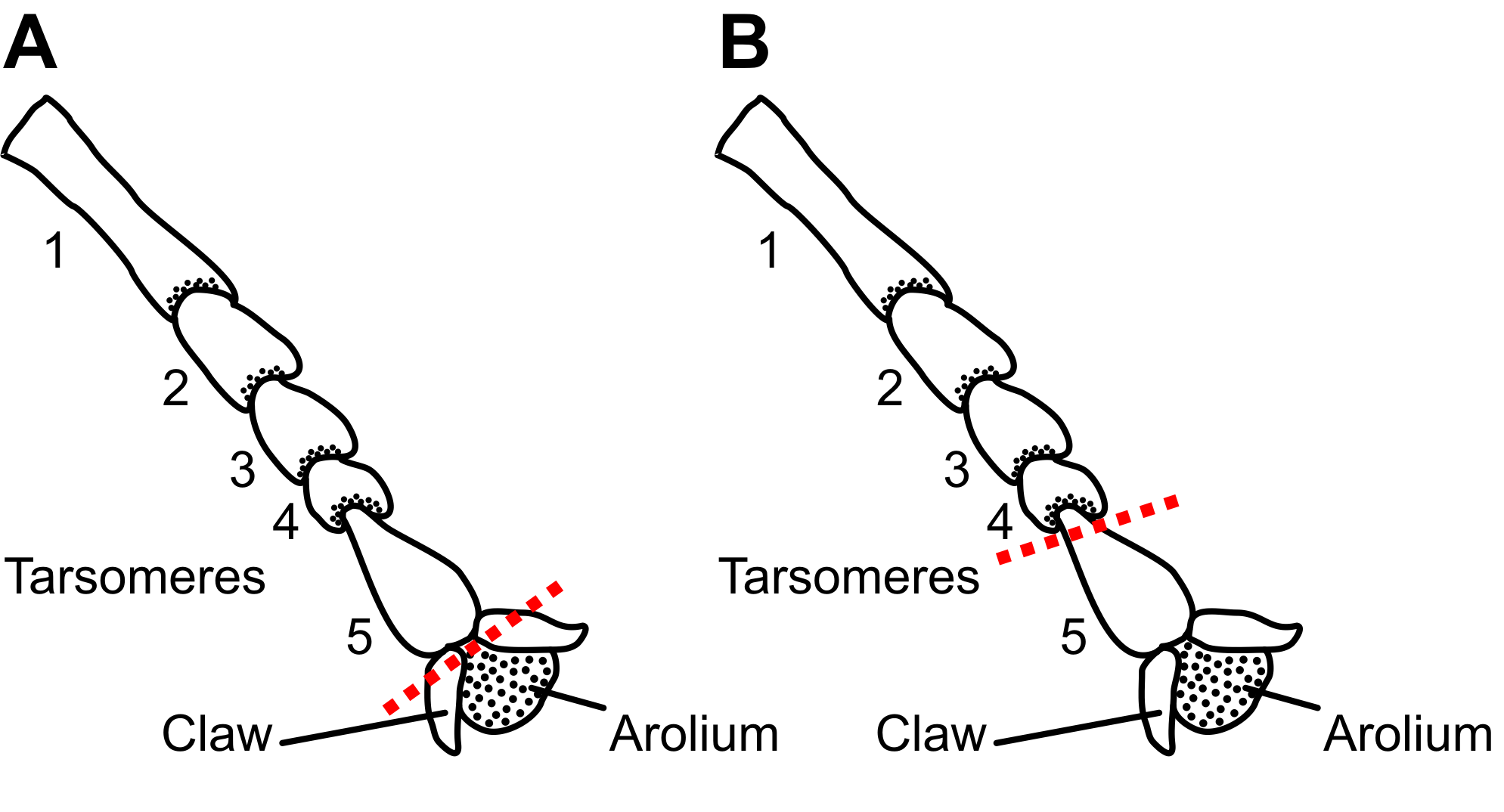

Supplement: Figure S1 — Tarsus manipulation experiments. A tarsus consists of five tarsomeres (numbered from proximal to distal) and the claws and the arolium. We either cut off the claw and the arolium (red line in A) or the entire fifth tarsomere (red line in B). Dots indicate unsclerosed membranes. (TIF) [file pone.0085321.s001.tif]

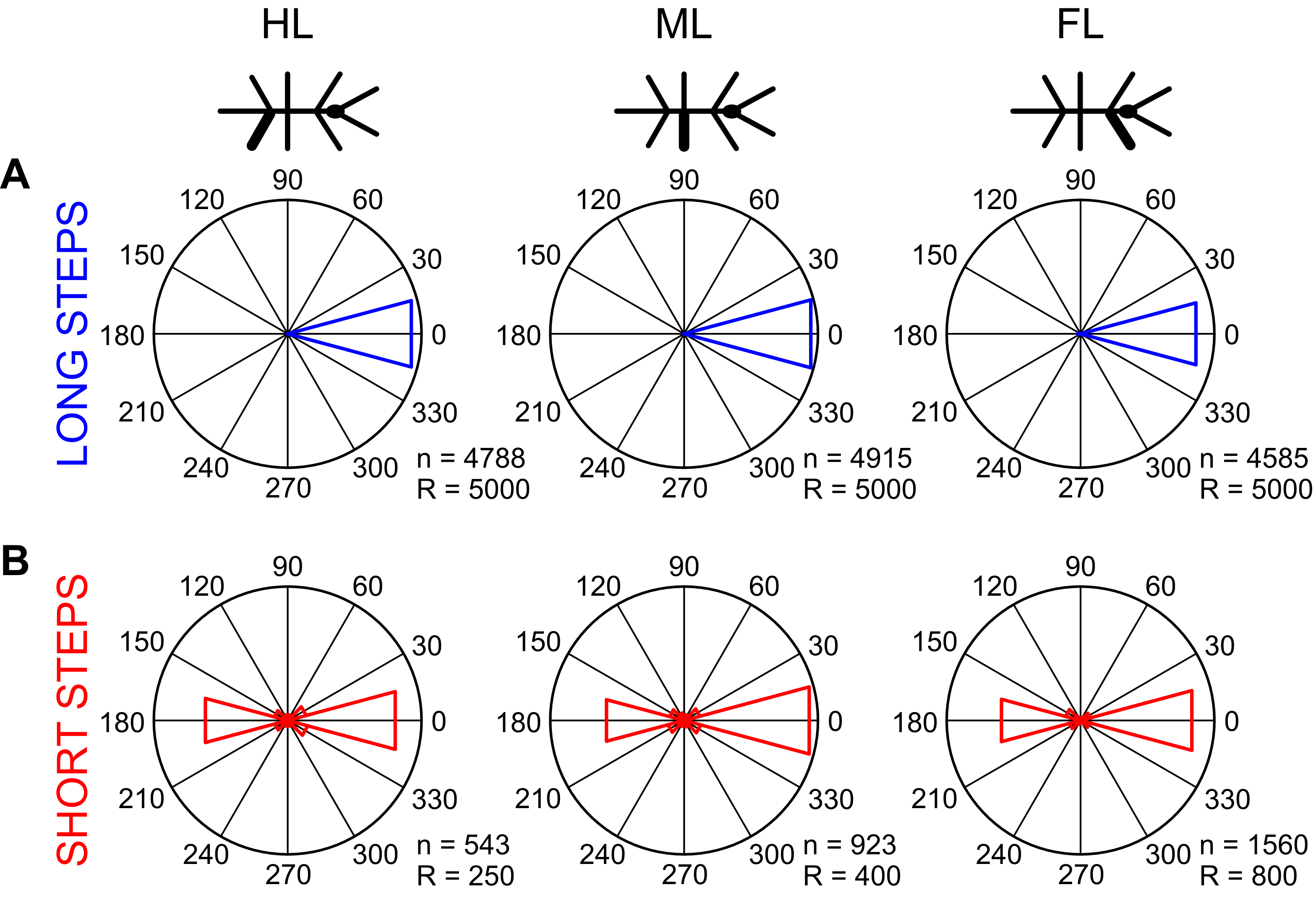

Supplement: Figure S2 — Limited position accuracy had no influence on the directional distribution of long and short steps. Same graphic details as in Figure 7A,B. To calculate the control of the direction angles, the x-value in body-fixed coordinates is kept and the y-value is replaced by random values of the range of the maximal jitter between the two thorax-fixed markers of all trials (= 0.6544). The number of steps (n) and the radius (R) of the outer circle are given on the lower right of each plot. (TIF) [file pone.0085321.s002.tif]

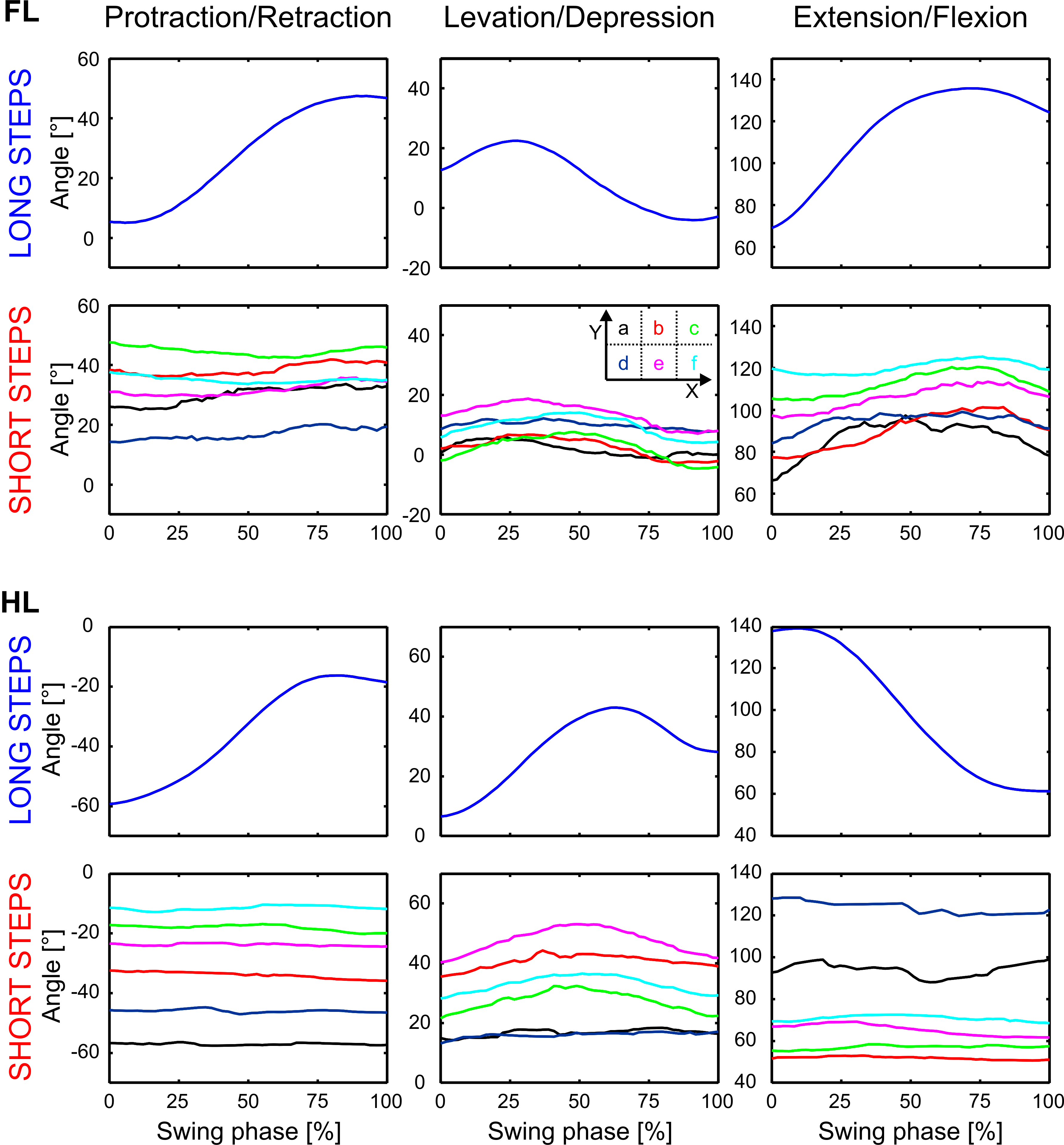

Supplement: Figure S3 — Joint angle time courses differ between FL (top) and HL (bottom), and between short steps (second and fourth row from top) and long steps (first and third row from top). Same plot details as in Figure 8, but for different leg types. The number of long steps was 4585 for FL and 4788 for HL. The numbers of short steps of the sub-samples are given in Table S1. (TIF) [file pone.0085321.s003.tif]
