# Supplementary material for: Insects Use Two Distinct Classes of Steps during Unrestrained Locomotion
Source: PLoS One. 2013 Dec 23;8(12):e85321. doi: 10.1371/journal.pone.0085321 (PMC3871641; doi:10.1371/journal.pone.0085321)
Supplement: Table S1 — Statistically relevant parameters of the mean swing directions of short steps, grouped according to their lift-off positions. Short steps were separated as described in Figure 7C into six sub-samples (a to f). The table shows the number of steps (n) of each sub-sample, which was used to calculate the mean swing direction (Figure 7C) and the median joint angle time course in Figure 8B (ML) and in Figure S2B,D (FL, HL). The level of significance (p) and the z-value correspond to the Rayleigh test used to calculate the mean swing directions. (DOCX) [file pone.0085321.s004.docx]

|  | FL | | | ML | | | HL | | |
| --- | --- | --- | --- | --- | --- | --- | --- | --- | --- |
| Lift-off | n | p | z | n | p | z | n | p | z |
| A | 276 | < 0.001 | 14.26 | 148 | < 0.001 | 24.23 | 96 | < 0.001 | 7.93 |
| B | 280 | < 0.001 | 20.17 | 158 | = 0.091 | 2.40 | 95 | = 0.018 | 3.99 |
| C | 224 | < 0.001 | 32.82 | 155 | = 0.206 | 1.58 | 80 | = 0.065 | 2.72 |
| D | 244 | < 0.001 | 11.23 | 159 | < 0.001 | 47.94 | 85 | < 0.001 | 25.93 |
| E | 240 | < 0.001 | 6.97 | 149 | < 0.001 | 8.44 | 86 | < 0.001 | 15.43 |
| F | 296 | = 0.735 | 0.31 | 153 | = 0.007 | 4.92 | 100 | < 0.001 | 7.96 |
